# The feasibility of using Microsoft Kinect v2 sensors during radiotherapy delivery

**DOI:** 10.1120/jacmp.v17i6.6377

**Published:** 2016-11-08

**Authors:** David M. Edmunds, Sophie E. Bashforth, Fatemeh Tahavori, Kevin Wells, Ellen M. Donovan

**Affiliations:** ^1^ Department of Physics The Royal Marsden NHS Foundation Trust Sutton UK; ^2^ Department of Physics Royal Holloway University of London Egham UK; ^3^ Centre for Vision Speech and Signal Processing University of Surrey Guildford UK

**Keywords:** radiotherapy, motion monitoring, sensors, electromagnetic interference

## Abstract

Consumer‐grade distance sensors, such as the Microsoft Kinect devices (v1 and v2), have been investigated for use as marker‐free motion monitoring systems for radiotherapy. The radiotherapy delivery environment is challenging for such sensors because of the proximity to electromagnetic interference (EMI) from the pulse forming network which fires the magnetron and electron gun of a linear accelerator (linac) during radiation delivery, as well as the requirement to operate them from the control area. This work investigated whether using Kinect v2 sensors as motion monitors was feasible during radiation delivery. Three sensors were used each with a 12 m USB 3.0 active cable which replaced the supplied 3 m USB 3.0 cable. Distance output data from the Kinect v2 sensors was recorded under four conditions of linac operation: (i) powered up only, (ii) pulse forming network operating with no radiation, (iii) pulse repetition frequency varied between 6 Hz and 400 Hz, (iv) dose rate varied between 50 and 1450 monitor units (MU) per minute. A solid water block was used as an object and imaged when static, moved in a set of steps from 0.6 m to 2.0 m from the sensor and moving dynamically in two sinusoidal‐like trajectories. Few additional image artifacts were observed and there was no impact on the tracking of the motion patterns (root mean squared accuracy of 1.4 and 1.1 mm, respectively). The sensors’ distance accuracy varied by 2.0 to 3.8 mm (1.2 to 1.4 mm post distance calibration) across the range measured; the precision was 1 mm. There was minimal effect from the EMI on the distance calibration data: 0 mm or 1 mm reported distance change (2 mm maximum change at one position). Kinect v2 sensors operated with 12 m USB 3.0 active cables appear robust to the radiotherapy treatment environment.

PACS number(s): 87.53 JW, 87.55 N‐, 87.63 L‐

## I. INTRODUCTION

As radiotherapy has become more conformal via developments such as intensity modulation, dealing with patient motion has been of increasing importance since highly conformal treatments are more sensitive to setup variations and organ motion. Approaches for dealing with patient motion range across a spectrum, from intrafraction tracking of implanted internal markers to the restriction of breathing motion^1^, ^2^ and have become more complex. Surface imaging has a role within motion management, and there is a range of commercial optical and infrared surface imaging such as VisionRT^(3)^ Varian Optical Surface Monitoring System (OSMS)^(4)^ NDI Polaris^(5)^ and C‐RAD Catalyst.^(6)^ Whilst these provide no information about the internal anatomy of the patient, they may be used in conjunction with ionizing radiation imaging to provide frequent or continuous monitoring throughout the radiation delivery, as they do not contribute to the radiation dose burden. Recently some groups have investigated surface monitoring for radiotherapy and PET imaging using consumer‐grade sensors designed for the entertainment industry — the Microsoft Kinect devices.^7^, ^8^, ^9^, ^10^, ^11^, ^12^, ^13^, ^14^, ^15^ The potential advantage of commercial off‐the‐shelf (COTS) devices is the low cost (less than ~$1,400 for a Kinect device and accessories compared to upwards of $250,000 for the commercial medical systems), thus enabling many sensors in many rooms, depending on the application.

Medical linear accelerators generate high‐energy, high‐dose‐rate radiation via a pulse forming network triggering an electron gun and a magnetron/klystron. Typical pulse repetition frequency (PRF) for 6 MV photon beam production is 400 Hz with a magnetron producing radiofrequency (RF) at GHz frequency. Equipment used in radiotherapy is subject to this source of electromagnetic interference (EMI). Hsu et al.^(16)^ demonstrated the important of assessing EMI for real‐time ultrasound tracking during radiotherapy delivery. Previous proof of concept reports of Kinect systems in radiotherapy have not provided any information or data on the effect of EMI due to the operation of the linear accelerator, or the need for USB 3.0 active (repeater) cables which are required to carry the signal from Kinect v2 sensors beyond 3 meters.^7^, ^8^, ^9^, ^10^, ^11^, ^12^ Robustness to EMI, and operation at a distance, are necessary for these sensors to be a feasible option for real‐time monitoring from the control area during radiation delivery for radiotherapy treatment. The purpose of this investigation is to determine the effect on Kinect v2 sensors, used with active (repeater) cables, of the radiotherapy treatment environment.

## II. MATERIALS AND METHODS

### A. Radiotherapy environment

An Elekta Synergy linear accelerator (Elekta Ltd, Crawley, UK) with photon beam energies of 4, 6, and 10 MV was used for all investigations. Standard pulse repetition frequencies (PRF) were 400 Hz at 4 and 6 Mv and 200 Hz at 10 MV; a variable dose rate function means that PRF may vary between 6 Hz and 400 Hz.

### B. Kinect v2 sensor with active cable

Kinect v2 sensors (Mocrosoft Corp., Redmond, WA) contain an infrared transmitter and receiver which use a time–of–flight technique to provide information about the distance of objects from the device, a high definition (HD) camcorder, and an array of microphones for positional sound detection.^(17)^ The distance data were used in these experiments. The Kinect v2 output is a 512×424 pixel array of reported distance in mm for each pixel, based on a manufacturer distance calibration, at a maximum frame rate of 30 frames per second (fps). The v2 sensors are supplied with a 3 m USB 3.0 cable. Extending USB 3.0 signals beyond 3 m is challenging because of the sensors’ high bandwidth asynchronous data transfers. FireNEX–ULK–12 Active Repeater cables (Newnex Technology Corp. Santa Clara, CA) were tested with the Kinect v2 sensors to investigate if distant operation from the linac control area was possible. Three sensors, each with its own 12 m active cable, were used in this study.

### C. Software

**Figure 1:**
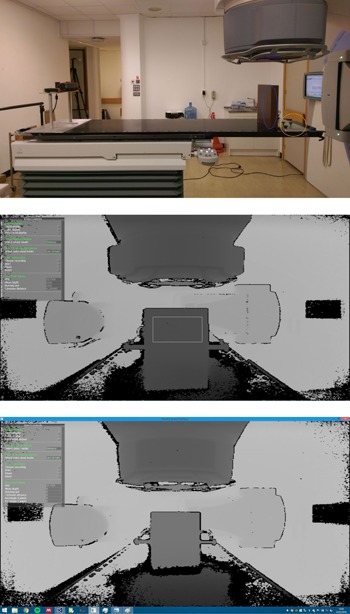
Experimental setup and effect of electromagnetic interference on the displayed images. (Upper panel) The sensor (red) is mounted on a custom stand manufactured in‐house. A thermometer is attached to the upper exhaust vent on the sensor. The Leica Disto D210 laser measure (green) is mounted securely next to the sensor, with the front plane lined up with the sensor. To the right, the solid water phantom (blue), used as the target, can be seen, along with the metal cross‐plate (orange) screwed into the couch which ensures the phantom is perpendicular to the sensor. (Middle panel) A depth camera view of a linac and solid water phantom on the treatment couch; darker objects are closer to the camera and lighter objects are further away. A region of interest (ROI) drawn by the user on the phantom is shown as a gray outline. The mean Kinect reported distance value of the pixels in this (ROI) in every frame was recorded to a file. Image was taken without the PFN operating. (Lower panel) Worse‐case image, with a PRF of 400 Hz. The circled region on the image indicates a region where additional image artifacts were observed.

The free Kinect for Windows Software Development Kit (SDK) 2.0^(17)^ was used to develop in–house software for capturing Kinect‐reported distance data and displaying these data as a grayscale image related to the distance of objects from the sensor (see Fig. 1).

### D. Monitoring of static and moving objects during radiation delivery

A 30×30cm solid water block phantom was the target object. A region of interest (ROI) was drawn on the image over the object and the software recorded the running average of the mean distance value over all pixels in the ROI in every frame at a rate of 30 frames per second. A Kinect v 2 sensor was fixed rigidly to the radiotherapy couch using an in‐house manufactured mount for the static measurements (see Fig. 1). A Leica Disto D210 laser measurement device (Leica Geosystems AG, St. Gallen, Switzerland) acted as the ground truth distance measure (ISO Standard 16331‐1 certified accuracy of ±1mm). Movement tracking was investigated with the phantom on an in‐house programmable motion platform which moved along three perpendicular Cartesian axes to follow a predefined motion pattern. The platform reported position to 1μm.

**Figure 2:**
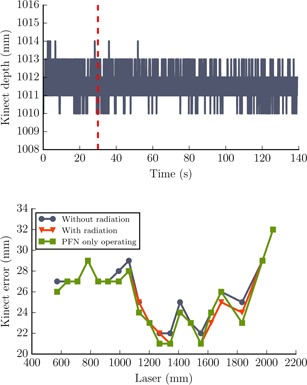
Effect of electromagnetic interference on Kinect‐reported distance output. (Upper panel) An example of Kinect‐reported distance to the stationary solid water block at a distance of 1.5 m from the sensor. The red dotted line indicates when radiation was switched on. The sensor output was unaffected by the operation of the PFN or the presence of radiation. (Lower panel) An example of a sequence of calibration measurements made without radiation, with the PFN firing at 400 Hz and with radiation on at each fixed distance. Differences between measurements are <1mm, maximum difference of 2 mm.

Kinect‐reported distance measurements are sensitive to the temperature of the device.^6^, ^18^ We confirmed this effect by imaging the solid water phantom at a fixed distance from the sensor continuously from switch on, and recording the sensor and ambient temperatures at 5 min intervals up to 60 min and every 30 min to 8 hrs after switch‐on. This was repeated at least five times over a three‐month period for each sensor. All data collection in these investigations were made after thermal stability was reached.

Data from the Kinect v2 sensors were collected with the linear accelerator in four states: (i) powered up only, (ii) with the pulse forming network and magnetron operating but no radiation production, (iii) with PRF 6, 12, 25, 50,100, 200, 400 Hz, and (iv) radiation delivery at

4 MV, 6 MV, 10 MV with and without the flattening filter and with dose rates of 50, 100, 200, 300, 400, 540 and 1250, 1450 monitor units (MU) per minute. The aim was to identify visually whether there were any observed effects and, if so, whether these were from the pulse chain electronics of the accelerator or the radiation delivery.

The solid water phantom was imaged when static at a distance of 1.5 m from the sensor. Then it was moved to a sequence of positions between 0.6 m and 2.0 m from the sensor to provide a distance calibration.^18^, ^19^ The distance and X, Y calibration process was repeated regularly for each Kinect over a four‐month period. Finally, the phantom was moved using the motion platform in two trajectories. The first trajectory was a flattened sinusoid with a peak‐to‐peak amplitude of 20 mm and a period of 12 s; the second was a sinusoid with a peak‐to‐peak amplitude of 20 mm and a period of 2 s.

The Kinect v2 sensor was designed for home entertainment under lighting conditions different from that of a radiotherapy treatment room. The reported distance of the phantom at 0.6, 1.0, and 1.5 m was recorded with the treatment room lighting varied from brightly lit, moderately lit, and with ambient light only from the linac console screens, lasers, and couch lighting. An RS 180–7133 lux meter (RS Components Ltd., Corby, UK) reported illuminances of 200, 9, and 0 lux, respectively.

## III. RESULTS

### A. Thermal and ambient lighting effects

Sensors’ temperatures increased until a plateau around 45 min and the reported distance changed by up to 6 mm towards the ground truth value then remained stable to 8 hrs post switch‐on. This occurred for all sensors regardless of the time of day or the ambient room temperature, and was consistent over the three‐month measurement period. There was no effect on the reported output from lighting conditions.

### B. Operating the sensor during radiation delivery

Three sensors were used in the experiments. Details are given for one, as the results were the same for the other two. The active cables used were placed in proximity to the gantry drum and electronics and along the room maze. There was no difference in the results. Undefined pixel values were returned around the edges of the frame and at the edges of objects in the baseline (linac not operating) images. Few image artifacts were observed when the pulse forming network was operating and when radiation was delivered. Figure 1 shows a typical image where the linac was not operating and the worst example observed when the PRF was 400 Hz. The percentage of undefined pixels in the image to total number of pixels showed a change of <1% across the PRF and MU ranges for an individual sensor (typical values per sensor 13% to 17%). Five repeat measurements at each PRF showed a variation <0.1%. The central 340×280 pixel region of the depth image was unaffected; the image size was 512×424 pixels.

**Figure 3:**
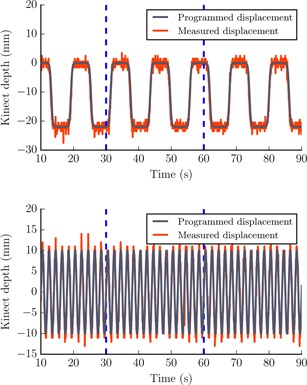
Effect of electromagnetic interference on Kinect output when tracking amoving object. The two programmed motion trajectories tracked by the Kinect v2. Blue dotted lines indicate radiation switched on and off at 30 and 60 s, respectively.

No effect on the Kinect‐reported distance output within the ROI of the static water phantom was observed when the PFN was operating or during radiation delivery. Variation in the distance calibration data was mostly 1 mm, with a maximum change of 2 mm (Fig. 2). The accuracy of the uncalibrated Kinect distance outputs was 2.0 to 3.7 mm across the three Kinect sensors over the object range 0.6 m to 2.0 m. A cubit fit to the data improved this to 1.2 to 1.4 mm if a distance calibration was carried out at each time of use. The relationship of Kinect‐reported distance to true distance formed a complex, but stable, pattern for all sensors (Fig. 2). This pattern was consistent with time for each sensor. There was a variation of 2 mm to 5 mm between sensors at each measurement point; a maximum variation of ±4mm was observed from datasets collected over four months. The standard deviation of output over an ROI of the flat object was 1.5 mm. The programmed motion trajectories were monitored with Kinect sensors with a root‐mean‐square (rms) error of 1.4 mm and 1.1 mm, respectively; there was no effect from EMI on the recorded outputs (Fig. 3).

## IV. DISCUSSION

This investigation of Kinect v2 sensors operated with FireNEX‐ULK‐12 Active Repeater cables demonstrated they can be operated remotely from a radiotherapy control room and whilst the pulse forming network is operating, with little effect on the output of the sensor, specifically within the central region of the image. To our knowledge, this has not been investigated or discussed in other work using Kinect v2 sensors. These characteristics are essential if these sensors are to have any feasible role in radiotherapy.

The measured distance accuracy of the Kinect v2 sensors is similar to that reported by others for both v1 and v2 sensors: several millimeters which can be reduced to <2mm or better with an appropriate correction.^13^, ^14^, ^18^, ^20^ Where systems are to be used for a clinical application, particularly if that involves monitoring small movements of a patient, it is important to determine the magnitude and impact of any EMI effect. This is well demonstrated in the analysis by Hsu et al.^(16)^ of ultrasound real‐time tracking in radiotherapy. The Kinect v2 sensors and the shielded extended USB 3.0 cables do not show any unwanted effect on the images, and the impact on the quantification of distance data was within the accuracy and precision of the devices.

Kinect sensors can identify the motion of phantoms and volunteers, particularly periodic respiratory motion.^8^, ^11^ Our future work includes a patient study where the Kinect v2 system will be assessed as an alternative to visual monitoring for a voluntary breathing hold method for breast cancer patients.^(21)^ Typical breath‐holding results in chest wall movement of 5 to 10 mm from the free‐breathing position; the Kinect v2 sensors have sufficient sensitivity to detect motion of this magnitude.

We have not presented any information on the lifetime of the sensor in a radiation environment. We have placed a Kinect v2 sensor within a clinical linear accelerator bunker with a set of thermoluminescent detectors (TLD) to monitor the dose. Sensor characteristics will be measured regularly over a long time frame to assess radiation damage. The low cost and ease of exchange of the sensors may mitigate any reduced lifetime due to radiation damage.

It is feasible to use Kinect v2 sensors with active (repeater) cables during radiotherapy delivery. Whilst unwanted image detail was observed, this was outside the central 45% of the field of view where a patient would be visualized. Data within this region were minimally affected, in both static and moving phantom situations. This latter is of most importance if the sensors are used in radiotherapy for setup assistance, to track breathing motion or their output used to gate a linear accelerator.

## ACKNOWLEDGMENTS

This report is independent research supported by the National Institute of Health Research (Career Development Fellowship, Dr Ellen Donovan, CDF‐2013‐06‐005). The views expressed in this publication are those of the author(s) and not necessarily those of the NHS, the National Institute for Health Research or the Department of Health. The work was undertaken in The Royal Marsden NHS Foundation Trust which receives a proportion of its funding from the NHS Executive. The authors acknowledge the NIHR Biomedical Research Centre at The Royal Marsden and the Institute of Cancer Research. The authors wish to thank Clive Long and Craig Cummings at the Institute of Cancer Research workshop for fabricating the custom sensor mount; Stephen Hamilton and Joseph Nurse for assistance with the radiation experiments; and Microsoft for three Kinect v2 sensors and accessories.

## COPYRIGHT

This work is licensed under a Creative Commons Attribution 3.0 Unported License.
